# A standardized workflow for kinetic metabolic model curation and dissemination

**DOI:** 10.1371/journal.pcbi.1014227

**Published:** 2026-04-20

**Authors:** Margaret Cook, Stella Anastasakis, Adel Heydarabadipour, Janis Shin, Diego Alba Burbano, James M. Carothers, Herbert M. Sauro

**Affiliations:** 1 Molecular Engineering and Sciences Institute, University of Washington, Seattle, Washington, United States of America; 2 Department of Chemical Engineering, University of Washington, Seattle, Washington, United States of America; 3 Department of Bioengineering, University of Washington, Seattle, Washington, United States of America; University of Maryland, Baltimore County, UNITED STATES OF AMERICA

## Abstract

Kinetic metabolic models provide invaluable insights into cellular metabolism, supporting applications in synthetic biology, metabolic engineering, and systems biology. However, reproducibility and utility of these models hinge on clear and rigorous documentation, standardized annotation, and accessible visualization. This paper presents a workflow for building, annotating, visualizing, and sharing kinetic metabolic models. Our method integrates community standards and open-source tools to ensure reproducibility, interoperability, and user accessibility. This procedure enables researchers to produce reusable and well-documented kinetic models, advancing their role as powerful tools in metabolic research.

## Author summary

In this work, we present a workflow for making computer models that simulate the dynamics of cellular metabolism, known as kinetic metabolic models, easier to understand, share, and build upon. These models help scientists study how biochemical systems process nutrients and energy, which is important for everything from developing new medicines to engineering microbes for sustainable production of materials. However, these models are most useful if others can easily reproduce them, understand what they represent, and use them in their own work. To address this, we outline a set of simple, effective methods for building, documenting, visualizing, and publishing these models. We show how to clearly describe what each part of the model does, create helpful visual diagrams, and share models in a way that others can easily access and reuse. Our goal is to make these powerful tools more transparent and collaborative, so that they can better support progress across biology, medicine, and biotechnology.

## Introduction

The optimization of biosynthetic pathways is a cornerstone of metabolic engineering, yet it remains a significant challenge due to the complexity and interconnectedness of living systems [1–3]. Metabolism is inherently dynamic, responding to changes in cellular environments, regulatory mechanisms, and external stimuli [4]. To approach the challenge of manipulating and optimizing these complex systems, researchers have turned to computational tools. Kinetic metabolic models, constructed using a system of ordinary differential equations, describe the temporal dynamics of metabolic networks. The integration of reaction-rate data, metabolite concentrations, and flux distributions enables the identification of promising metabolic engineering targets and rapid pathway prototyping [5]. However, the construction of kinetic models is a resource-intensive process, requiring significant time and effort to curate data, define parameters, and validate simulations [6]. Despite the time and effort going into these carefully constructed models, there are very few guidelines on how to standardize and publish these models [7]. Thus, many of these models remain inaccessible, incomplete, or inconsistent across publications, leading to duplicated efforts which hinder progress in the metabolic engineering field [8] and a significant loss of intellectual effort

**Fig 1:**
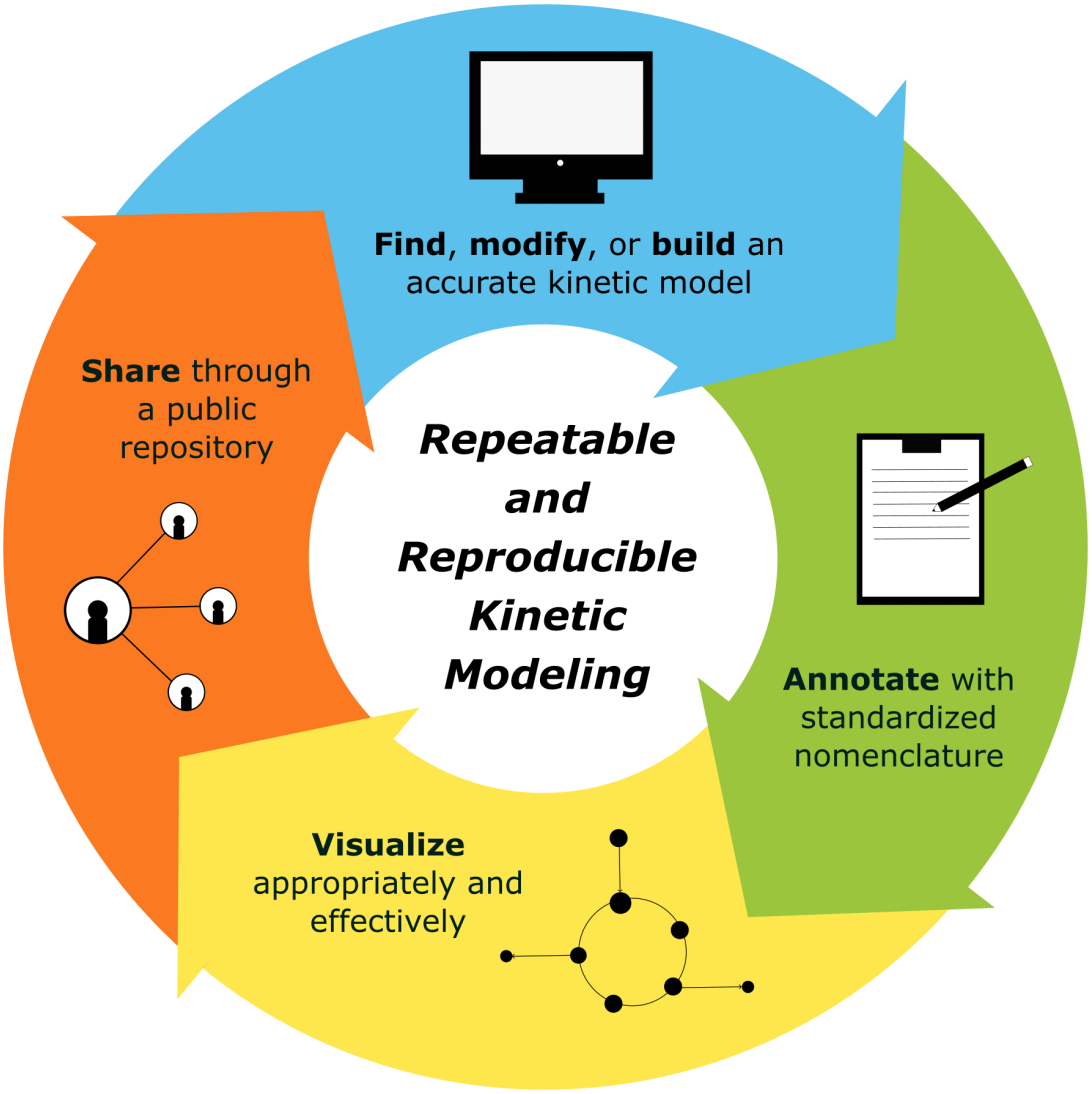
Workflow for employing best practices in kinetic modeling. By annotating and sharing relevant files and visualization directions through open-source channels, researchers can contribute to a more repeatable and reproducible set of kinetic models.

To accelerate research and foster collaboration, it is essential to move toward standardized, open-source, and publicly available models. Standardization ensures that models are interoperable, reproducible, and easy to build upon, reducing the need for researchers to repeatedly reinvent foundational components [9]. Best practices for model publication, therefore, should prioritize thorough annotation, insightful visualization, and adherence to standardized formats (Fig 1).

In this paper, we present a methodological framework for developing and publishing kinetic models illustrated through a case study describing metabolite dynamics in a crude cell lysate [10]. Crude lysates are a type of cell-free protein synthesis system in which microbial cells are lysed to release a mixture of proteins and substrates capable of performing transcription and translation. The lysate in this example is derived from *E. coli* cells, and has been used to prototype multi-enzyme pathways for metabolic engineering [11–14]. While numerous well-characterized kinetic models exist for *E. coli* metabolism [15–17], they cannot be directly applied to answer questions regarding cell-free lysates due to key differences in enzyme abundances, compartmentalization, and cofactor regeneration. The included case study demonstrates how existing models can be leveraged and adapted to address new biological contexts, highlighting the practical aspects of model reuse. Importantly, it also shows how adherence to steps in our workflow, such as standardized annotation, versioned model sharing, and clear documentation, enables this kind of reuse in the first place. We close the loop by showing how our own exemplar model is constructed to be similarly reusable and extensible for future work. By walking through the construction, annotation, simulation, and dissemination of this model, we highlight key steps for effective model sharing:

1. **Finding and editing or building a model:** There exist numerous kinetic models already described and published in repositories like BioModels [18], yet they may not always fully address the specific needs of a study. Reusing and adjusting these models when possible is preferable to building an entirely new model, and using tools which allow for direct manipulation of the model to incorporate additional reactions or adjust parameters makes this easier. If existing models are insufficient, building a new kinetic model will likely involve parsing stoichiometric and kinetic information from databases like BRENDA [19], assembling this reaction information into Synthetic Biology Markup Language (SBML) [20], and fitting [21] the preliminary model to experimental data, if available.
2. **Model annotation**: Ensuring that every model component is clearly defined, with consistent and comprehensive annotations, minimizes ambiguity and facilitates reuse by others. Specialized, user-defined nomenclature should be accompanied by standardized annotations to prevent the loss of information or misinterpretation.
3. **Informative and reproducible visualization**: Visualization of reaction fluxes or other salient data plays a crucial role in model interpretation and dissemination. Linking models to standardized visualization workflows, especially those compatible with SBML, helps ensure consistency and ease of understanding. Including the exact programmatic instructions to obtain the published visual representation of the model allows researchers to replicate and modify these representations easily.
4. **Open-source sharing**: To maximize impact, kinetic models should be published in a standardized format such as SBML, along with all relevant data, simulation code, and metadata. These materials should be deposited in a recognized model repository to provide context and ensure transparency, reproducibility, and accessibility. These steps empower the research community to build on existing models rather than starting from scratch.

By emphasizing these principles, we provide a concrete method for improving the usability and reproducibility of kinetic metabolic models, ultimately advancing the collective knowledge in systems and synthetic biology.

## Methods

### Find and edit or build a model

Kinetic models are widely used in metabolic engineering and studies of metabolism and regulation more broadly, but they can also be quite time-consuming to construct. Science progresses through iterative improvement, so it is more productive and time-efficient to reuse pre-existing models and tools which others have created than to remake the models entirely.

### Identify suitable existing models

To minimize redundancy and accelerate model development, a literature search should first be performed to determine whether a pertinent model already exists. Established model repositories should also be queried, such as BioModels [19] which contain kinetic models, or other model repositories that may not have kinetic information, but serve as useful starting points via highly curated genome-scale models (GSMs), such as BiGG Models [22] and Metabolic Atlas [23] (Table 1A). These models are generally written in standard SBML (Table 1B) which encourages interoperability and reproducibility, while avoiding the challenges of reconstructing a model that may be built on non-standard or poorly recorded design choices, making it unclear or difficult to implement.

**Table 1:** Resources available to achieve best practices in kinetic modeling.

Method/Tool	Link	Features
**A. Model Repositories**		
Literature search	N/A	Access to the full study and context behind models; many models are unpublished elsewhere. However, parsing papers is time-consuming, and models may not follow standardized formats, include downloadable files, or be free from errors.
BioModels	http://biomodels.net/	Open-source and curated with extensive metadata, standardized formats, and filtering by organism, approach, and tags. Primarily an SBML based repository.
BiGG models	http://bigg.ucsd.edu/	Highly standardized genome-scale models with Escher visualization, extensive annotations, and multiple download formats. Focuses on whole-species metabolism, limiting availability and subsystem-specific models.
Kbase	https://www.kbase.us/	Genome scale models only, stored as SBML. Open-source and collaborative platform with extensive functionality including data integration, annotation with RAST, and flux balance analysis.
Metabolic Atlas	https://metabolicatlas.org/	Detailed annotations, organized by compartments, with high-quality visualizations and data overlay for comparison. However, it has a limited scope and can be challenging to navigate.
ModelDB	https://modeldb.science/	Database of computational neuroscience models. Hosts models in many formats and programming languages.
ModelSeed	https://modelseed.org/	Genome scale metabolic model reconstruction pipeline for plants and plant-microbe interactions. Downloadable as SBML format, Model SEED format, or LP format.
Physiome model repository	https://models.physiomeproject.org/	Simple browser interface with graphical previews and easy downloads. Models are primarily in CellML format, search functionality is basic, and naming conventions can be unclear.
***B. Software data formats to describe models***		
SBML	https://sbml.org/	Supports the representation of reaction-based models and mathematical models where necessary.
CellML	https://www.cellml.org/	Component based format for mathematical models, suited for multi-scale processes but less common for metabolic models because it doesn’t explicitly represent reaction networks.
***C. Text based model definition languages***		
Antimony	https://github.com/sys-bio/antimony	Human-readable, script-based language for defining and modifying SBML models; supports modular model building but requires conversion to SBML for simulations.
PySCeS Model Description Language	https://pysces.sourceforge.net/	Human-readable, text-based format for defining models which run directly, and exclusively, in PySCeS. Once loaded into PySCeS can export SBML.
SBML-shorthand	https://github.com/darrenjw/sbml-sh	Minimal, human-readable text format for creating SBML models; designed for concise specification of reactions, parameters, and species without verbose XML. Requires conversion to SBML for simulations.
***D. Converters***		
Tellurium	https://github.com/sys-bio/tellurium	Able to interconvert between SBML and Antimony. Packaged with extensive model simulation capabilities.
AWE	https://sys-bio.github.io/AntimonyEditor/	Able to interconvert between SBML and Antimony. Is a useful tool for writing out a model. Additional features like rate law insertion, annotation creation, and syntax highlighting and error detection.
MakeSBML	https://sys-bio.github.io/makesbml/	Simple web-based interconversion tool between SBML and Antimony. Is a useful tool for writing out a model and can load models directly from BioModels.
***E. Databases describing stoichiometric chemical reactions***		
KEGG	https://www.genome.jp/kegg/	Comprehensive, standardized reaction database with enzyme classifications and modules; lacks kinetic parameters but is easy to parse (No SBML support).
MetaCyc/BioCyc	https://metacyc.org/ https://biocyc.org/	Detailed enzyme-reaction links, directionality, inhibitors, and kinetic data; requires a subscription for high usage and is more difficult to parse Offers SBML file importer and exporter.
BRENDA	https://www.brenda-enzymes.org/	Extensive reaction and kinetic data, sortable by organism; supports automated parsing via tools like BRENDApy.
Expasy	https://www.expasy.org/	Simple format listing enzymes and reactions without additional details.
ExploreEnz	https://www.enzyme-database.org/	Minimalist enzyme-reaction database, easy to read and parse.
IUBMB	https://iubmb.org/	Basic enzyme-reaction listings, similar to ExploreEnz, with a simple structure for parsing.
***F. Databases containing kinetic parameters***		
BRENDA	https://www.brenda-enzymes.org/	Extensive kinetic data for substrates, products, and inhibitors; sortable by organism; data from papers may have inconsistent units and context; supports parsing via BRENDApy.
SABIO-RK	http://sabio.h-its.org/	Kinetic data with filtering by enzyme type, pH, temperature, and source; provides units and experimental context. Includes rate laws.
Literature search	N/A	Time-consuming; values may not match your system but come with experimental context.
Collecting experimental data	N/A	System-specific and accurate but requires lab access and can be time-intensive.
***G. Selection of model simulators***		
AMICI	https://github.com/AMICI-dev/AMICI	A C++-based simulator with Python and MATLAB interfaces. Specializes in ODE-based simulation and parameter estimation using sensitivities and gradient-based optimization.
COBRApy	https://opencobra.github.io/cobrapy/	Python package for constraint-based modeling, including flux balance analysis (FBA) and metabolic network optimization, widely used for studying metabolism and synthetic biology.
COPASI	https://copasi.org/	GUI-supported tool for simulating biochemical networks using deterministic (ODE), stochastic, and hybrid methods, with extensive parameter fitting and sensitivity analysis.
JWSOnline	https://jjj.bio.vu.nl/	Web-based platform for easy access to curated kinetic models, allowing interactive simulation and parameter modifications without local installation.
libRoadRunner through Tellurium	https://www.libroadrunner.org/	Python based modeling environment which supports both ODE, metabolic control analysis (MCA), stochastic, and constraint-based metabolic models via COBRApy. Interactive and user-friendly.
PySCeS	https://pysces.sourceforge.net/	Python library focused on steady-state analysis, metabolic control analysis (MCA), and time-course simulations for biochemical systems.
pySB	https://pysb.org/	Python framework for rule-based modeling of biochemical networks, suited for signaling pathways and complex reaction systems required rule-based specifications.
VCell	https://vcell.org/	Modeling environment which can be used through the web interface or via a desktop client. Useful for researchers interested in modeling cellular processes with spatial components or for those working with microscopy data.
Web Iridium	https://sys-bio.github.io/WebIridium/	Web-based platform for simulating kinetic metabolic models with an intuitive GUI for parameter adjustment, time-course and steady-state analysis, and customizable plots. Supports SBML and graphical export for downstream use.
***H. Model annotators***		
AWE	https://sys-bio.github.io/AntimonyEditor/	Allows for very precise annotation of each species; requires a large amount of manual effort and would be time consuming for large models.
Antotate	https://github.com/carothersresearch/Antotate	Automated and requires very little manual effort, but can make mistakes and requires proofreading.
***I. Model visualization***		
CellDesigner	https://www.celldesigner.org/	Takes a state transition diagram and supports model creation, simulation, and database integration. Desktop GUI implemented in Java, SBML compliant. Uses its own non-standard format (software-specific extension of SBML) for visualization.
		
Cytoscape	https://cytoscape.org/	Allows users to visually encode table data as network properties like color and size, which can be customized using Styles in the Control Panel. 2D layout of biochemical networks with a variety of layout algorithms. Java desktop application. Supports numerous file formats (SBML, SBGN, SIF, etc.)
Newt	https://newteditor.org/	Offers interactive diagramming, semantic validation, support for experimental data overlays, and advanced complexity management capabilities. Extension of Cytoscape, web-based UI. Style information stored in the.nwt file format.
PathVisio	https://pathvisio.org/	Modular design separates pathway view and data model, allowing for efficient experimental data visualization and statistical analysis using GenMAPP and MAPPFinder. Desktop Java-based tool. Uses Graphical Pathway Markup Language (GPML) file format, manipulated using libGPML.
Escher	https://escher.github.io/	Semi-automated pathway design integrating proteomics, metabolomics, and fluxomics data. Web-based tool with Python and Jupyter extensions. Integrates BiGG formats. Saved with JSON file format. To build a map, the model must be in COBRA format, or you can convert SBML to Escher. Works best for smaller models (less than 200 reactions) due to manual curation. Conversion to SBML requires external SBML Java app.
Vanted	https://cls.uni-konstanz.de/software/vanted/	Enables the superimposition of multi-experiment -omics data on networks, direct correlation analysis, and machine learning-based clustering. Java-based desktop tool to map experimental data onto network visualizations. Offers plug-ins for flux analysis and exploration of large metabolic models. Supports multiple file formats for export of visualization data (SGML, KGML, SBGN-ML etc.)
SBMLNetwork	https://github.com/sys-bio/SBMLNetwork	Reproducible, standard-based visualization through employing SBML Layout and Render packages to embed visualization data directly inside an SBML file. Force-directed autolayout algorithm for modeling of intricate networks without existing layouts. Intuitive command-line API to allow users to interact with the visuals of SBML models and seamlessly integrate it into their workflows.
***J. Model publishing***		
BioModels	http://biomodels.net/	Multiple model formats accepted (Primarily SBML, but has some CellML, MATLAB, Mathematica, R, C++ models). Extensive model curation procedure. Upon manuscript publication, authors request the public release of the model.
BiGG Models	http://bigg.ucsd.edu/	Metabolites linked to external databases (KEGG, PubChem, etc.); Model must be published in a peer-reviewed journal and use BiGG ID convention.
Physiome Model Repository	https://models.physiomeproject.org/	Users create a Git workspace to store all files for each model, which can then be published on the PMR database
ModelDB	https://modeldb.science/	Relatively simple submission process. The submitted model must be affiliated with a peer-reviewed article to be made public. Permits models made using any simulation software and programming language.

Once candidate models are identified, they should be evaluated for suitability with your specific system and research goals. A few key questions should be considered: (1) Does the model cover your system’s biological scope and can it address core research questions? (2) Are kinetic parameters and initial conditions clearly defined and biologically plausible? (3) Is the model in a standardized, editable format? (4) Has the model been validated or cited in peer-reviewed studies? Answering these questions helps ensure the model will be smoothly integrated into your own research and adaptable for your purposes.

### Convert and edit existing models

**Fig 2:**
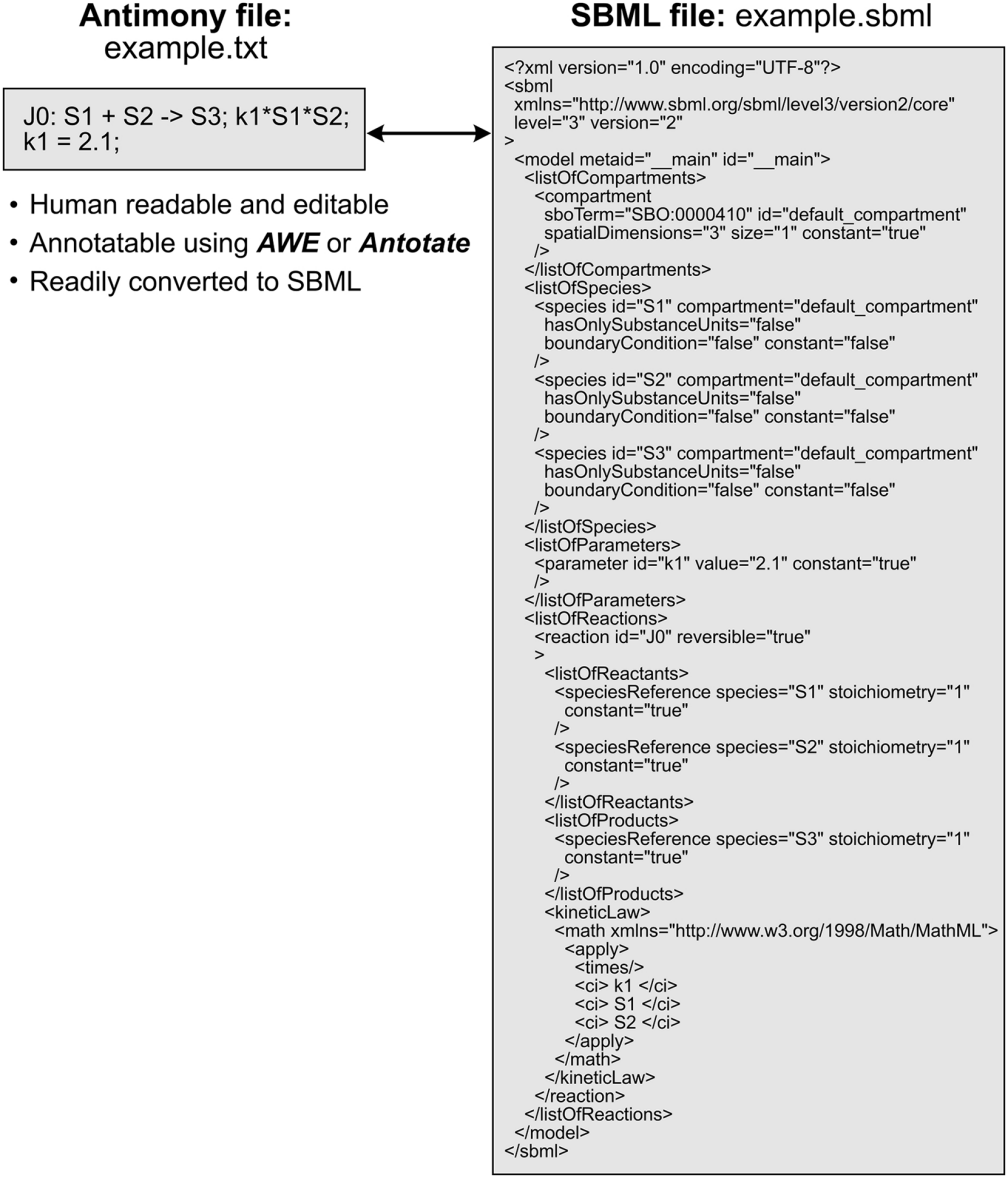
Antimony and SBML interconversion. While it is still best practice to include the XML version of a published kinetic model, it is more practically useful for future users of the model to work with a human-readable format. Antimony is one such example of a human-readable model definition language which readily interconverts with the machine-readable model definition language, SBML.

Once existing models have been identified and deemed suitable, they should be downloaded and customized by adding or removing reactions or modifying kinetic rates and parameters. The simplest way to modify a model’s structure is to first convert the computer readable version of the model (i.e., XML) to a human-readable, text-based format (Table 1C) using a converter (Table 1D). For instance, the Python package Tellurium [24] is able to convert SBML format, which is not human-readable, to Antimony. MakeSBML is a web-based tool that can interconvert SBML and Antimony [25]. Antimony is a human-readable model definition language that makes it easier to construct and annotate models by displaying reactions and their rates in a clear, intuitive format (Fig 2). Once the model is in Antimony format, or another text-based model definition language, users can intuitively edit the document in any text editor, removing the need for specialized knowledge of markup language and making customization more accessible and efficient [26]. If a published model includes annotations which describe the species and reactions present, it may be significantly easier to interpret and repurpose [27,28]. BiGG models, for instance, use standardized BiGG IDs for metabolites and enzymes, minimizing ambiguity. In BioModels, another highly curated model repository, over 90% of the models are annotated in some capacity, but only 15% of these models are fully annotated, with the remainder ranging widely in coverage [29]. In other less curated databases or models only published in affiliation with a manuscript, the quantity and quality of annotations lessens further. In such cases it is recommended that the user of the model utilizes annotation tools, which are further described in “Model annotation”, to improve its legibility and interpretability.

### Construct a new model

**Fig 3:**
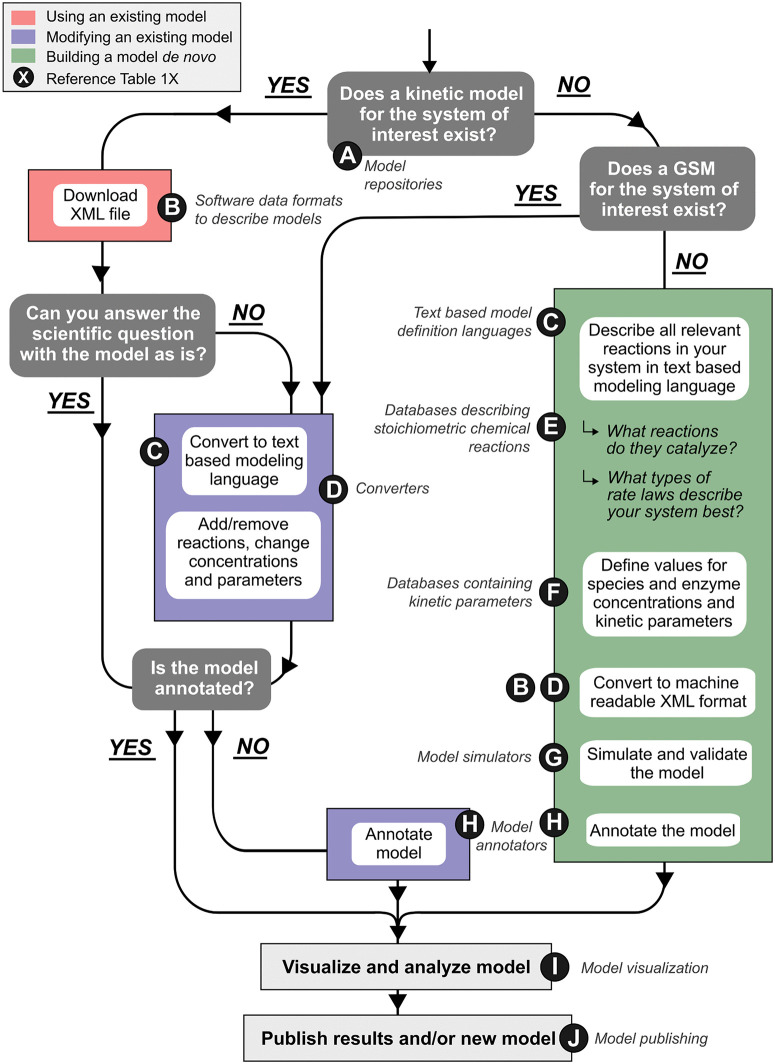
Workflow for constructing, modifying, or using kinetic models of biological systems. The flowchart guides users through decision points and actions based on whether a model for the system of interest already exists and whether it can answer a specific scientific question. Pathways include using existing models (red), modifying existing models (purple), or building new models *de novo* (green). Key steps include converting between formats, annotating models, simulating and validating, and ultimately analyzing and publishing results. Letters correspond to subsections of Table 1 describing relevant resources and tools.

If the system of interest does not have a published kinetic model available, one can be built *de novo*. This involves determining the appropriate reaction network followed by parameterizing the rate laws with empirically based values. These techniques for building a model can all be applied to existing models, kinetic or steady state, for modification or refinement (Fig 3).

Whether constructed from scratch or adapted from prior work, tailoring a model to a new system requires the addition of reactions, adjustment of parameters, and validation against experimental data. To guide this process, we outline below a generalized workflow for kinetic model construction:

1. **Define the scope:** Before constructing a kinetic model, it is critical to consider the biological context the model is meant to represent and the goals of the experiments. Will the kinetic model describe metabolism in a whole cell, a subcellular compartment, a cell-free extract, or the interaction of metabolically different cells? At this stage, it’s also useful to consider the desired level of granularity. Can reactions be lumped together to simplify certain modules? The choice of kinetic formalism [30] (e.g., Michaelis–Menten, reversible modular rate laws, linlog, power law, or hybrid approaches) should reflect both the biological complexity of the reactions being modeled and the availability of data. More detailed mechanistic rate laws can capture reversibility, multi-substrate reactions, regulation, and cooperativity, but introduce additional parameters that may be difficult to identify. Simpler or approximate formalisms reduce parameterization burden and can improve identifiability, at the cost of mechanistic interpretability. Defining the scope upfront helps avoid overfitting, underparameterization, or unnecessary complexity, and ensures the model is tailored to the questions it is meant to address. Where available, curated genome-scale metabolic models provide a strong starting point to define the stoichiometric skeleton, as they undergo extensive manual curation and are typically tested for mass and flux consistently under steady-state assumptions. When constructing kinetic models from GSMs, care should be taken to ensure that simplifications do not introduce mass, redox, or energy flows that would be infeasible in the parent model. At the same time, kinetic models rarely benefit from retaining the full complexity of a GSM. Model reduction strategies, including the removal of inactive or redundant reactions or coarse-graining/lumping, allow modelers to remove extraneous detail while maintaining consistency with genome-scale behavior and supporting reliable kinetic analysis [31]. In cases where genome-scale metabolic models are unavailable or incomplete, metabolites and reactions may be assembled using databases such as KEGG [32,33], MetaCyc [34], or BioCyc [35] (Table 1E). Once the reactions in the network are determined, write them out in Antimony or another text based model language with their rate laws.
2. **Parameterize the model:** Set initial values for chemical species, determine the kinetic parameters, and define them in the model. The number and type of parameters required depend strongly on the chosen kinetic formalism: mechanistic rate laws that account for reversibility, multiple substrates or products, or regulation introduce substantially more parameters than approximate formalisms such as linlog or power-law models. If experimental kinetic data is available, finding the parameter values can be done by fitting a curve through the experimental data points [36]. If the kinetic law is simple, such as a power law, fitting can be done using Excel. For more complex kinetic equations, such as the Michaelis-Menten rate law, using a Python package such as lmfit [37] to fit the parameters is one option, but other software options exist, such as pyPESTO [38]. Typically, even if one has access to some kinetic data for the system, comprehensive parameterization may be limited by experimental, financial, or biological constraints. In such cases, parameter values can be obtained through databases such as KEGG or BRENDA (Table 1F) [19]. Additionally, machine learning tools have been developed which are able to predict initial values for kinetic parameters (K_M_ and K_cat_ values) provided with a protein sequence and SMILES [39] string of the corresponding substrate [40,41]. It is worth noting that many enzyme characterization assays are performed *in vitro, and ki*netic parameters measured in such conditions may not accurately reflect effective *in vivo* behavior [42–44] due to factors such as molecular crowding, enzyme organization, substrate channeling, or spatial heterogeneity. Changes in metabolic state, including transcriptomic or proteomic shifts, can also alter effective enzyme activities. Kinetic models can also incorporate non-metabolite factors that influence rates, such as transcriptional and translational regulation, light, temperature, or other environmental cues. These influences can be represented through additional reactions, modifiers, or factors that adjust reaction parameters in response to the relevant input. Parameter reuse and fitting should therefore consider how these state-dependent effects impact reaction rates in the specific biological context. Regardless of the source, parameter uncertainty should be considered explicitly, as these uncertainties propagate into model predictions.
3. **Validate the model:** Once the model has been fully constructed (all chemical reactions, participating metabolites, initial concentrations, kinetic rate laws, and corresponding parameter values) and defined, one should validate the model’s predictions with experimental observations. Data used for parameterization and validation may include direct measurements such as metabolite concentrations, enzyme abundances, or reaction rates obtained from *in vitro* assays, cell extracts, or whole-cell experiments. In many cases, indirect measurements, such as fluxes inferred from isotope labeling experiments, uptake and secretion rates, or steady-state flux balance analyses, can also be used to constrain or evaluate model behavior.

Parameterization may rely on time-course data to capture transient dynamics, steady-state measurements to constrain fluxes and concentrations under defined conditions, or a combination of both, depending on the modeling goals and data availability. Simulating the model can be done through Web Iridium [45], Tellurium, or other simulators described in Table 1G. If the model’s output does not align with experimental data, iterative refinement is typically needed. This may involve adjusting parameter values, testing alternative rate laws with possible allosteric regulation, modifying initial conditions, or revising the reaction network.

To assess model robustness and reduce the risk of overfitting, validation strategies such as sensitivity analysis, parameter ensemble sampling, identifiability analysis, and comparison against independent datasets or perturbation experiments can be employed. Together, these analyses help ensure that model predictions are both quantitatively reasonable and biologically interpretable

### Annotate metabolic models

Data annotation in kinetic models bridges the gap between personalized naming conventions and standardized, universally recognized identifiers. While researchers may use unique naming schemes during model construction (i.e., Glucose abbreviated as Glc, G, Dextrose, etc.), proper annotation ensures these designations are linked to well-characterized references in public databases. This is vital for models intended for publication and sharing, as it promotes consistency, reproducibility, and ease of use for the broader scientific community.

As described earlier, Antimony is a text-based model definition language which allows for simpler construction and annotation. Its intuitive syntax allows users to define internal species names, assign display names for visualizations, and, importantly, link species to external databases such as ChEBI [46], KEGG, and SMILES with the addition of a few simple lines of text (lines 1550–1675 in S4 File). These annotations provide an objective and unambiguous reference for each species, making the model comprehensible and reusable by others. Once annotations are incorporated into an Antimony file, they are seamlessly transferred to the corresponding Systems Biology Markup Language (SBML) file during conversion.

In practice, tools like Antimony Web Editor (AWE) [47], also available as the desktop extension VSCode-Antimony [48] (Table 1H), allow users to interactively annotate species. AWE aids users in drafting their kinetic model in Antimony with helpful color coding, has the ability to catch syntax errors, translates an Antimony file into SBML format, and allows for annotation with up to nine databases. Right click on a species and create an annotation by choosing the database of interest (e.g., ChEBI, UniProt, etc.) and search for the identifier which most clearly describes that species. This will add lines of text at the bottom of the Antimony document which encode the annotation and get ported seamlessly into SBML format. This method works very well for models with few numbers of species. However, in larger models it can become burdensome to manually annotate all of the species.

For larger models, where manual annotation becomes time-consuming, automated tools can assist. For example, the open-source tool Antotate [49] supports bulk annotation of Antimony models by suggesting likely matches for each species based on public databases such as KEGG or MetaCyc. While automated annotations require proofreading, tools like this can significantly reduce the manual burden and help enforce standardized vocabulary across a model.

For reproducibility and accessibility, it is essential to publish the model as an annotated SBML file. This ensures that other researchers can readily interpret, modify, and build upon the model with minimal barriers. By adopting this practice, we can standardize the sharing of kinetic metabolic models and enhance their utility within the scientific community. In the future, consistent and thorough annotation of published models will enable modular model development. This will allow researchers to quickly review existing models and seamlessly integrate standardized reaction components to accelerate prototyping.

### Visualize models effectively

Visualizing kinetic models as stoichiometric networks offers a powerful way to communicate insights and share findings with the broader community. Depicting network topology and overlaying dynamic behavior enhances understanding of complex model features. A broad range of software tools and visualization data formats have been developed for representing biological pathways (examples of diagram editors and pathway databases are listed in Table 1I). However, despite the extensive array of available tools, the interoperability and reproducibility of model visualization remain as major challenges. Many tools use tool-specific formats for storing visualization data, and common standards are only partially or inconsistently implemented. As a result, pathway maps created in one application cannot be faithfully reproduced or imported into another, which undermines reliable sharing of visual layouts alongside model content and complicates collaborative workflows [50]. To address this, we recommend embedding visual layout information directly within the model file using a standardized format, such as SBML.

SBML’s Layout [20] and Render [51] extensions store pathway layout and graphical styling information within the same SBML file, which unifies model content, visual layout, and styling data under a single, tool-agnostic standard. This consolidation removes the need for external graphics files that often hinder interoperability and reproducibility, because inside the unified document, every visual element is explicitly linked to its corresponding model component. With model elements and their visual counterparts co-located, tools can also reference the same identifiers to overlay dynamic data, such as concentrations and fluxes, directly onto the network topology without additional manual mapping. Further, coordinates from one map can be easily reused in a separate map of similar metabolic pathways. To generate standardized pathway diagrams, we suggest SBMLNetwork [52], a Python tool that produces visualizations fully compliant with the SBML Layout and Render standard.

An additional option widely used by the modeling community is Escher, a web-based tool for constructing and visualizing metabolic maps. Escher facilitates reproducible visualization by generating a JSON file which stores layout and styling instructions, which can be shared and reloaded across platforms. While these visualization instructions are not directly embedded in the model file like with SBMLNetwork, Escher’s maturity, availability of pre-existing map templates, and extensive use in published models make it a practical choice for some applications.

To ensure that visualizations effectively support analysis and interpretation, especially for large kinetic models with complex dynamics, we recommend following established practices that improve clarity, consistency, and interpretability [53]. These include selecting appropriate layouts, maintaining visual balance, and using color, size, and labels to encode relevant information. In the following section, we provide specific guidance on how to implement these visualization practices to generate reproducible, interpretable pathway diagrams suited for publication, communication, and reuse.

### Generate a clear model layout

The layout of reactions and their associated entities in a kinetic model is crucial for readability and comprehension. It forms the foundation for conveying a pathway’s biochemical logic, so it must reflect the inherent relationships and progression of reactions. When well organized, the layout allows viewers to trace the process intuitively and effortlessly understand how the system’s components interact. A layout strategy should be selected based on the model’s complexity and the user’s objectives.

As a first strategy, we recommend using automated layouts. Hierarchical or force-directed algorithms, such as those available in SBMLNetwork [54], can be used to generate an initial layout. Species and reaction visuals are distributed over the canvas to minimize node overlap and edge crossings, resulting in a clean, first-pass layout that spares users from manually specifying every coordinate. This automated step is especially useful for newly built or small models without existing visualizations and provides an immediate view of the network’s structure.

**Fig 4:**
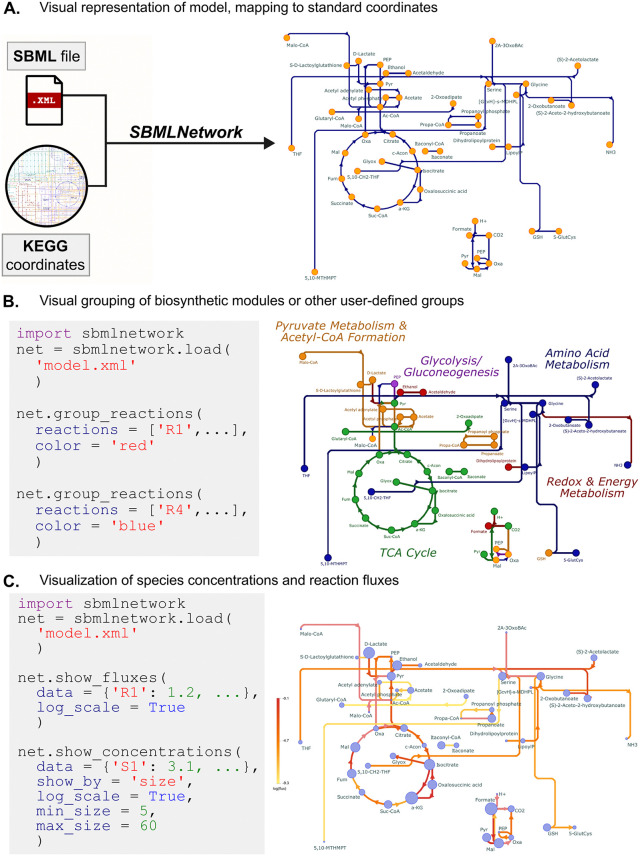
Strategies for effective visualization of data from kinetic models. **(A)** Choosing a graphical representation of a kinetic model which follows community standards enables users and collaborators to more easily draw conclusions and observe trends. SBMLNetwork is able to accept predefined coordinates, from KEGG, for example, and map the corresponding species in the kinetic model onto those positions. **(B)** Color-coding species nodes and reaction curves is an effective way to distinguish reaction groups and highlight important parts of a model. The example shown includes pyruvate metabolism & Acetyl-CoA formation (orange), glycolysis/gluconeogenesis (purple), amino acid metabolism (blue), redox and energy metabolism (red), and the TCA cycle (green). **(C)** SBMLNetwork and other visualization tools allow integration of quantitative data such as reaction fluxes and species concentrations. Reaction fluxes and species concentrations are both displayed on a logarithmic scale, using a yellow to red colorbar and node size, respectively.

Another effective strategy is to adopt predefined layouts that have become standard in the literature for well-established metabolic modules. Using resources such as KEGG Metabolic Pathways [32,33] and Escher-generated metabolic maps, layout coordinates can be extracted and reused to recreate community-accepted network topologies, such as those for glycolysis or the citric acid cycle. SBMLNetwork supports this process by enabling manual adjustment of element coordinates, which allows researchers to seamlessly replicate and adapt established pathway maps. This approach simplifies the task of mapping new networks while preserving familiar and interpretable visual structures, reducing manual effort and promoting consistency with published conventions (Fig 4A).

A third strategy is to manually curate the layout when automated or predefined options do not meet specific visualization goals or capture key pathway features. In this approach, ensuring clarity and an intuitive structure that reflects biochemical logic is essential. For example, a top-to-bottom or left-to-right orientation can highlight directional metabolite flow, whereas a circular arrangement may better represent cyclic pathways. To maintain readability, position elements to avoid overlap and minimize curve crossings so that each interaction remains distinct. In dense networks, duplicating certain nodes, particularly ubiquitous cofactors, can reduce visual clutter and preserve local connectivity [55]. Adding visual compartments, graphical boundaries that represent biological spaces such as the cytoplasm, periplasm, or extracellular environment, further enhances spatial context by grouping components according to their biological locations. These steps make manual curation a powerful and flexible option for refining pathway diagrams.

### Apply styling principles for interpretability

Styling specifies how model components are rendered through deliberate choices of size, shape, color, line weight, and labels. These choices turn a merely accurate layout into a clear, visually coherent graphic that highlights biological meaning and makes kinetic behavior easier to interpret. To design informative and intuitive model diagrams, the following core styling principles are recommended. Marker shapes play a central role in distinguishing molecular entities and interactions within a pathway diagram. Assigning a dedicated shape to each molecular type keeps the model visually organized and allows viewers to differentiate components quickly, even in complex networks. Interaction types can likewise be encoded with arrowhead geometries: distinct tips for binding, activation, or inhibition enable readers to identify relationship types at a glance. SBGN (Systems Biology Graphical Notation) [56] provides a set of shape conventions one can use to set all model element shapes. Here, consistency is crucial; applying the same shape conventions throughout the entire diagram reinforces clarity and ensures that the visual language remains informative. Color is another powerful means of embedding quantitative context into a pathway diagram. Use a graded palette to color species nodes to represent dynamic properties, such as gradients in metabolite concentrations, enzyme activities, or gene-expression levels, using saturated hues for higher values and lighter tints for lower ones. Applying the same color gradient to reaction curves, where line color indicates flux, creates a consistent visual scale for both metabolite loads and reaction flows. This unified scheme allows readers to quickly assess relative magnitudes and identify patterns or outliers that might be missed in numerical tables. Pathway modules or functional groupings can be highlighted by assigning a shared color and line weight to selected reactions and their associated species (Fig 4B). Tools such as SBMLNetwork support automatic color assignments for grouped reactions. Adjusting node dimensions is an effective way to convey importance or magnitude within a network. In dense models, uniformly reducing node size prevents overcrowding and preserves topological clarity, whereas larger nodes in simpler diagrams maintain visual balance and direct the viewer’s focus. Selectively enlarging key species, such as a target product in a metabolic-engineering pathway, emphasizes elements of particular biological or experimental interest. When applying data-driven sizing, appropriate bounds are essential: excessively small nodes may disappear, while overly large nodes can obscure neighboring elements. For example, dynamic scaling of node size can be used to represent metabolite concentrations on a logarithmic scale, enabling intuitive recognition of concentration ranges (Fig 4C).

Line weight can likewise serve as a quantitative cue for reaction magnitude: wider edges denote higher flux or rate, whereas narrower ones indicate lower activity. Scaling widths in proportion to flux enables viewers to distinguish major pathways from minor routes at a glance. To make kinetic models interpretable, clear and consistent labeling should be employed. Each visual element should be distinctly labeled to allow viewers to readily identify components and follow the network logic. Labels should generally adhere to a uniform font style, font size, and placement across the diagram, and should be positioned as close as possible to the entity they represent to promote visual coherence and reduce ambiguity. To emphasize critical nodes, such as pathway endpoints, regulatory hubs, or engineered targets, selectively increasing font size can help draw attention without disrupting the overall layout. This approach must be applied with care: overly large labels may obscure nearby elements or compromise the diagram’s clarity. Additional semantic cues can be introduced through typographic styling. For example, using *italics* for enzyme names helps distinguish them from metabolites or other molecular species, while **boldface** can highlight elements of particular biological or experimental interest. To ensure effective model diagrams, styling elements should be applied in a balanced and coordinated manner. Avoid overusing any single visual channel, as this can overwhelm the viewer and obscure key biological insights. A controlled and well-proportioned usage of each component helps ensure that key features are emphasized without introducing unnecessary visual noise. We also recommend the coordinated use of multiple visual channels to reinforce meaning. For example, representing reaction flux using both color intensity and line weight offers a multimodal depiction of pathway activity, which makes differences in model throughput immediately apparent. Similarly, using a label in a larger font alongside a unified color scheme can help visually segment a specific region of the network and draw readers’ attention to a functional module or pathway section. Overall, each styling element should be applied with intentionality. Thoughtful integration of multiple visual elements such as shape, color, and size ensures key model information stands out, while preserving interpretability and focus.

### Support reuse with open model sharing

Once a kinetic model has been carefully constructed, thoroughly annotated, and effectively visualized, it is ready to be shared with the broader community. Open-source sharing has significantly accelerated scientific innovation. Prominent examples include Python and Linux, an open-source programming language and operating kernel, respectively, which have driven rapid technological advancement across a wide variety of fields. Open-source sharing is important for collaboration within the scientific community. Users can add features and fix bugs, resulting in higher quality software. Instead of waiting for a single developer to add a requested feature, the user can create the feature themselves. With a multitude of users sharing improvements and updates, problems can be solved with a variety of creative solutions. Further, software improvements can occur more rapidly by leveraging the collective knowledge of the community.

Not only does open-source sharing promote widespread collaboration, it also reduces barriers to reproducibility in science. For instance, the computing platform MATLAB requires a paid subscription to use which can be a barrier for accessibility and discourages broader use. Researchers without MATLAB subscriptions cannot replicate results or build upon published work. Open-source languages like Python have grown rapidly thanks to widespread community collaboration, enabling continuous development through new libraries and tools. In contrast, MATLAB’s closed-source nature limits contributions from the broader community and restricts its accessibility. Similarly, Mathematica, a symbolic mathematical computation software, requires a paid license to download, making models built and published with its code inaccessible to the public and difficult to recreate. In general, models published as programmatic code, for example as raw differential equations, tend to be very difficult to reuse [57,58].

When publishing a model, researchers should follow the FAIR (Findable, Accessible, Interoperable, and Reusable) and CURE (Credible, Understandable, Reproducible, and Extensible) principles [59,60] to enhance discoverability, usability, reproducibility. Our recommended model publication workflow includes the following steps: (1) provide model files in standardized formats such as SBML and Antimony in the published paper; (2) embed annotation and visualization specifications directly in the model file, or provide accompanying code and files in a supplemental repository; (3) make associated experimental data publicly available and explicitly document all computational experiments, including which variables were modified and how model results were generated; and (4) upload the model to a public repository for easier access by the broader scientific community. Experimental data and supporting files can be hosted on platforms such as FAIRDOMHub [61] or Zenodo [62]. Locations to publish kinetic models include BioModels, BiGG Models, and PMR, which all have different submission standards (Table 1J). These practices promote reproducible, accessible, and high-quality models. Adhering to the FAIR and CURE principles for data ensures models are effectively and efficiently exchanged between collaborators for further innovation and discovery across research projects.

## Results

Having outlined the methodological steps for effective kinetic modeling, we now demonstrate their application through a case study of crude *E. coli* lysate metabolism. This example illustrates how existing models can be leveraged and adapted to address new research questions while remaining reusable for future work.

### Literature survey and model reuse

As a first step, we performed both a literature survey and a search of model repositories to assess the landscape of potentially relevant models. Although no complete, reusable, and fully in-scope model of crude lysate metabolism was available in standard repositories, we identified several related resources in the literature. One model describes the protein synthesis dynamics in cell-free systems and another covers lysate metabolism but is implemented in MATLAB, a non-open source platform which presents barriers to reuse and interoperability [63,64]. In addition, *E. coli* is extremely well-studied, and numerous kinetic models of its metabolism have already been developed [15–17]. These models, while not directly usable in our context, served as valuable starting points for developing a lysate-specific framework.

Rather than constructing a model entirely from scratch, we therefore leveraged an existing, well-annotated kinetic model of *E. coli* metabolism [15] as the foundation for our cell-free lysate model. This model provided a curated reaction network with strong overlap to the endogenous metabolism of crude lysates as well as reusable estimates for kinetic parameters.

### Assembly, parameterization, and validation

We then adapted the model to the specific biological context of a cell-free lysate by comparing the enzymes present in the curated model to proteomics data from our lysate preparation [65]. This allowed us to systematically prune reactions catalyzed by enzymes absent or poorly represented in the lysate, while retaining core metabolic functions that were experimentally supported. Heterologous enzymes relevant to our pathway engineering goals were added by identifying their catalyzed reactions in KEGG and formulating rate laws based on their substrates and products. To balance biological realism with the practical need to select rate laws for each reaction, we employed a common modular rate law [66], which captures reaction reversibility and multi-substrate kinetics while maintaining a consistent parameter structure across the network. These equations were written in Antimony, and initial guesses for kinetic parameters were obtained from BRENDA (S1 File).

**Fig 5:**
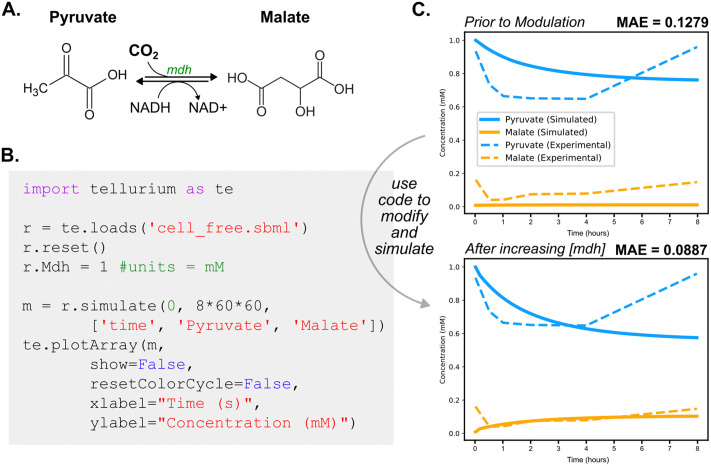
Simulation-based validation of a kinetic model using Tellurium. **(A)** The reaction catalyzed by *malate dehydrogenase* (*mdh*), interconverting pyruvate and malate. **(B)** Example Python code using Tellurium to load the model, modify enzyme concentration (e.g., [*mdh*]), simulate time-course dynamics, and plot metabolite concentrations. **(C)** Comparison of simulated versus experimental metabolite concentrations over eight hours, before (top) and after (bottom) increasing [mdh] to 1 mM. Model performance improves upon enzyme adjustment, reducing mean absolute error (MAE). This iterative process of simulation and refinement ensures the model better captures observed biological behavior.

The parameterized model was then fit to experimental data. For this case study, implemented in Antimony and simulated in Tellurium, we focused on the dynamics of pyruvate and malate over an eight-hour period (Fig 5A), for which experimental time-course data were available [13]. Initial simulations showed an underestimation of pyruvate consumption and an absence of malate production compared to the experimental data. This discrepancy highlights a common challenge in kinetic modeling: parameters derived from purified protein measurements or database values often require context-specific adjustment to account for effective enzyme activity in complex biological environments.

To improve the model’s performance, we increased the concentration of *malate dehydrogenase* (EC 1.1.1.38), which interconverts pyruvate and malate via oxaloacetate, to 1 mM (Fig 5B). This adjustment led to improved agreement with the data, capturing both the early pyruvate consumption and the emergence of malate (Fig 5C). Such targeted refinements can reflect emergent effects such as enzyme colocalization, substrate channeling, or altered effective kinetics in cell-free systems, rather than literal changes in enzyme abundance. This type of targeted refinement may be iterated to minimize error, with care taken to avoid overfitting and maintain empirical validity and biological plausibility. Model validation strategies such as sensitivity analysis, parameter ensemble sampling, and comparison to independent experimental conditions can help distinguish compensatory parameter fitting from biologically meaningful corrections.

This example illustrates how kinetic models are commonly parameterized and validated in practice, particularly when comprehensive and condition-matched kinetic datasets are unavailable. These challenges are not unique to this case study and are often more pronounced in non-model organisms, where both kinetic parameters and experimental measurements such as spent-media profiles may be sparse. In such settings, model reduction and coarse-graining strategies, together with informed parameter estimation based on available data or predictive tools, can still yield useful and testable models [67]. Beyond model construction, effective model development also depends on how the model is annotated, visualized, and shared. In addition to accurately simulating biological phenomena, models must also be transparent, interpretable, and reusable.

### Annotation

Annotation is not only important for publishing a model; it is also essential for building one efficiently. In our workflow, we leveraged existing annotated *E. coli* models to identify and extract reactions, enzymes, and metabolites relevant to our cell-free system. Annotations made it possible to match components across models and ensure consistency in naming and stoichiometry. Without standardized annotation, the process of repurposing existing models would require significantly more manual curation and would be more error-prone. For our case study, the model comprised 57 reactions, making annotation in AWE impractical. Instead, we used Antotate to generate initial annotations, which we then proofread and amended to produce final annotated Antimony (S2 File) and SBML files (S3 File).

### Visualization

Visualization plays a complementary role by making complex networks easier to interpret. In our case study, there was no single Escher map encompassing all of the reactions in our model, so we instead used the coordinates from KEGG’s *E. coli* pathway map as the basis for model layout with SBMLNetwork (Fig 4A). To aid interpretation, we highlighted biosynthetic modules (Fig 4B) by grouping them and applying uniform hues with enlarged, consistently styled labels, visually separating each module from surrounding pathways. Additionally, we overlaid reaction fluxes onto the network using a graded color palette on reaction curves (Fig 4C). These design choices demonstrate how careful use of layout and color can clarify pathway structure and encode quantitative meaning in an intuitive manner.

### Sharing

Finally, we emphasized model sharing as a central outcome of the workflow. Our cell-free model contains both annotations and visualization instructions embedded in the SBML file, enabling straightforward reuse and ensuring that others can reproduce the layout and semantics without additional effort. Packaging the model in this way makes it simple to distribute alongside a publication and maximizes its potential as a reusable and extensible resource for future work.

Taken together, this case study illustrates the full arc of our workflow: from identifying existing resources, through adapting and parameterizing a model to a new biological context, to annotating, visualizing, and sharing the final product. By demonstrating each step in the context of crude *E. coli* lysate metabolism, we highlight how methodological best practices translate into concrete outcomes, and how reusable, well-documented models can accelerate future research across diverse systems.

## Discussion

Kinetic metabolic models offer insight into addressing complex biological questions. However, the lack of standardized practices for creating and sharing these models results in inconsistencies and limited reproducibility that hinder substantial progress in the modeling field. To address this, we presented a practical and adaptable workflow for building and disseminating kinetic metabolic models. This workflow includes four comprehensive steps for kinetic modeling: 1) how to create a model, or find and edit an existing one; 2) proper model annotation for clarity and interoperability; 3) visualization strategies for effective scientific communication; and 4) the use of open-source platforms for sharing a model.

Firstly, an existing model can be found from a model repository, and edited to suit the user’s needs. Alternatively, a user can generate a model *de novo* using Antimony to define the reactions and components of the system in a human-readable format. Models should then be thoroughly annotated using tools such as AWE or Antotate. Informative, accessible visualization can seamlessly be integrated into the model using SBMLNetwork. Finally, the model can be published through recognized model repositories such as BioModels and accompanying code can be shared on open-source platforms such as GitHub, enabling future use by other researchers.

While this workflow is illustrated using a well-curated model organism, many of its steps are designed to remain applicable in settings where metabolic knowledge and experimental data are sparse. In non-model organisms, kinetic parameters and uptake or secretion rates may be incompletely characterized; in such cases, models can be parameterized using bounded estimates from homologous enzymes, relaxed flux constraints, and ensemble or sensitivity-based approaches to identify parameters that most strongly influence system behavior. Similarly, when spent-media data are unavailable, approximate exchange bounds informed by literature values or thermodynamic feasibility can be used and iteratively refined as additional data become available. Additionally, transfer learning is emerging as a promising strategy for accelerating kinetic metabolic model development by training machine learning models on existing metabolic models for related systems. This approach could be valuable for non-model organisms with limited structural information, such as genome annotations, and kinetic data [68–70].

Kinetic models can also capture changes in metabolic state, including shifts in enzyme abundance or activity driven by transcriptional, translational, or environmental factors such as light or temperature. These influences can be incorporated by adding reactions, modifiers, or factors that adjust reaction parameters in response to the relevant input. Explicitly representing such state-dependent effects allows models to remain biologically meaningful across conditions, even when comprehensive data is unavailable.

By standardizing these steps, we aim to lower the barrier to entry for new modelers and promote the development of reproducible, extensible kinetic models that can be broadly applied across metabolic engineering, systems biology, and synthetic biology research.

## Supporting information

S1 File Unannotated antimony file of model. (TXT)

S2 File Antimony file of model preliminarily annotated with Antotate. (TXT)

S3 File Confidence metrics from Antotate. First tab is the direct output of Antotate. Second tab is the manually corrected output of Antotate.
(XLSX)

S4 File Fully annotated Antimony file of model. (TXT)

S5 File SBML file of model with annotations. (SBML)

S6 File SBML file of model with annotations and SBMLNetwork visualization instructions. (SBML)
